# Development of a sustainable route for the production of high‐fructose syrup from the polyfructan inulin

**DOI:** 10.1049/nbt2.12031

**Published:** 2021-03-22

**Authors:** Kongkona Saikia, Hridya Radhakrishnan, Abiram Karanam Rathankumar, Siva Gokul Senthil Kumar, Shravani Kalita, Jenet George, Sivanesan Subramanian, Vaidyanathan Vinoth Kumar

**Affiliations:** ^1^ School of Bioengineering Integrated Bioprocessing Laboratory SRM Institute of Science and Technology Chennai India; ^2^ Department of Applied Science and Technology AC Tech Anna University Chennai India

## Abstract

The authors used mesoporous silica microspheres as a support for the immobilization of inulinase from *Aspergillus brasiliensis* MTCC 1344 by the process of cross‐linking. Under optimized operating conditions of pH 6.0, particle/enzyme ratio of 2.0:1.0 and glutaraldehyde concentration of 7 mM, a maximum immobilization yield of 90.7% was obtained after a cross‐linking time of 12.25 h. Subsequently, the cross‐linked inulinase was utilized for the hydrolysis of 5% inulin, and a maximum fructose concentration of 31.7 g/L was achieved under the optimum conditions of pH 6.0 and temperature 60°C in 3 h. Furthermore, on performing reusability studies during inulin hydrolysis, it was observed that the immobilized inulinase could be reused up to 10 subsequent cycles of hydrolysis, thus providing a facile and commercially attractive process of high‐fructose syrup production.

## 1 INTRODUCTION

High‐fructose syrup (HFS) has increasingly solicited industrial importance over the years as an ingredient in food and pharmaceuticals. As it is 1.2 to 1.6 times sweeter than conventional sucrose, fructose is widely used in the food industry as a sweetener with flavour‐enhancing properties [1]. It also serves various non‐food applications and has been widely utilized for the production of bulk chemicals. Recently, the dehydration of fructose has been extensively explored for the production of the important platform chemical, 5‐hydroxymethylfurfural, which can be utilized for the production of an array of chemical building blocks [2]. The conventional method for the commercial production of fructose is the isomerization of glucose, mediated by the enzyme isomerase [1]. However, the enzymatic isomerization of glucose produces an equimolar mixture of the two carbohydrates, which situates an economic constraint in the separation process. Moreover, being a direct competitor in the food chain, the utilization of glucose for fructose production is less preferable [3]. In this context, the hydrolysis of a fructose‐rich polysaccharide, like inulin, can confer a promising alternative for the commercial production of fructose.

Inulin is a polyfructan consisting of fructose units where the fructosyl units are linked by linkages terminating with a glucose unit [4]. Generally, fructose can be produced from inulin either by enzymatic or chemical hydrolysis. However, chemical hydrolysis leads to the undesirable degradation of inulin, which may hinder the production of highly pure fructose. Thus, alternatively, inulin hydrolysis by the action of inulinase proves to be an effective route for fructose production [5, 6, 7]. A number of works have been reported towards the utilization of free inulinase from various microbial sources for the production of fructose from inulin by hydrolyzing the *β*‐1,2‐fructan links in inulin. Some of the widely explored sources of inulinase include *Aspergillus ficuum* [8], *Aspergillus niger* [9], *Kluyveromyces marxianus* [10], *Fusarium oxysporium* [11] and *Candida guilliermondii* [12]. The utilization of inulinase provided a single‐step hydrolysis process for inulin which can yield a high fructose concentration of 95–96% [13]. However, the involvement of free inulinase in a batch reaction is not feasible as it cannot be used in consecutive batches of inulin hydrolysis [5]. Moreover, the risk of microbial contamination of the substrate dictates the requirement of a hydrolysis process that operates at high temperatures, thus annulling the growth of microorganisms [14]. Consequently, these setbacks urge the development of a thermally and operationally stable enzymatic system for inulin hydrolysis, which can be overcome by immobilization of inulinase. The utilization of immobilized inulinase has garnered much attention as immobilization delivers a facile route for the recovery of the immobilized enzyme, thus reducing the overall cost of the process. Moreover, immobilization of enzyme allows the usage of a high density of the enzyme in the reaction, besides providing thermal as well as operational stability to the immobilized enzyme and also increasing the shelf life [5, 15]. Previously, inulinase was immobilized via various techniques amongst which covalent binding and cross‐linking were mostly preferred. Even though covalent binding is generally preferred for enzyme immobilization, it limits the maximum enzyme loading capacity due to the immobilization of only mono‐layer of the enzyme molecules on the support [15]. Thus, to overcome the demerits, inulinase was cross linked on to the silica microspheres support. Cross‐linking of the enzyme leads to little desorption and the cross‐linking agents activate the functionalized supports which increase the efficiency of the immobilization strategy [15].

In recent years, various supporting agents like chitosan, ion‐exchange resins etc. were used for the immobilization of inulinase [16]. However, the utilization of nano‐particles as a support for enzyme immobilization is widely accepted, since they offer high surface area to volume ratio, thus resulting in high enzyme loading. Amongst the various nano‐sized supports, silica nanoparticles have garnered much attention as they are chemically and mechanically stable, have uniform shape and provide small diffusion limitation [15]. The porous structure of these particles protects the enzyme from the harsh reaction conditions like high temperatures, extreme pH and the presence of inhibitors, creating an ideal micro‐environment for the enzyme. The pore sizes of silica particles are comparable to the diameter of enzymes, owing to which these particles have garnered much attention as supports for enzyme immobilization [17]. Even though several works have been done on the application of mesoporous silica for immobilization of enzymes, there are limited studies done on the utilization of these particles as a support for the immobilization of the industrially important enzyme, inulinase. These particles exhibit high surface area due to its size, are chemically inert and biologically inactive and, therefore, do not participate or intervene significantly in any chemical or biochemical process [18]. Further, due to the high surface‐to‐volume ratio and low diffusion limitation, the mesoporous silica microspheres increase the enzyme loading per unit mass of the support [15]. Moreover, considering the commercial value of HFS and its conventional production from corn through multistep processes, inulinase provides a single‐step solution for the green production of HFS to reduce complexity, time and cost.

Thus, considering the advantages of mesoporous silica microspheres and the conceivable commercial application of inulinase in the food industry, this study aimed at the sustainable development of a robust immobilized inulinase system on functionalized silica microspheres with improved reusability for the production of HFS. Various immobilization parameters were optimized to apprehend their effect on the efficiency of immobilization. The cross‐linked inulinase was subsequently utilized for hydrolysis of inulin and the associated process parameters were optimized for maximum yield.

## 2 MATERIALS AND METHODS

### 2.1 Chemicals and microorganism

Inulin and fructose of analytical grade were purchased from Sisco Research Laboratories Pvt. Ltd., Chennai, India. Fructose (≥99%), used as a standard for high‐performance liquid chromatographic analysis, was procured from Sigma‐Aldrich (St. Louis, MO, USA). All the other chemicals used were of analytical grade and were obtained from Sisco Research Laboratories Pvt. Ltd., Chennai, India. D‐Fructose Assay Kit was purchased from Megazyme (Bray, Ireland). Mesoporous silica microspheres were provided by Materium Innovations (Granby, QC, Canada). *Aspergillus brasiliensis* MTCC 1344, procured from Microbial Type Culture Collection (MTCC), Punjab, India, was maintained in the modified Czapeck Dox medium for the production of the enzyme inulinase [19]. Inulinase from *A. brasiliensis* was harvested by centrifuging the culture at 3000 rpm for 20 min. The crude inulinase was then partially purified by a three‐phase partitioning technique according to our previous work [19], by saturating the crude inulinase with 60% ammonium sulphate and adding equal volumes of *t*‐butanol. After 2 h of incubation, the obtained interfacial layer was separated and resuspended in phosphate buffer (pH 6.0), to make a final concentration of 500 U/g with 10.2‐fold purity as per our previous report [17], and utilized for the immobilization studies.

### 2.2 Characterization of mesoporous silica microspheres

The elementary composition of the mesoporous silica microspheres was determined by the energy disruptive X‐ray spectrometer (EDS). The analysis of the surface functional groups of the mesoporous silica microspheres was performed in a Cary 660 fourier‐transform infrared spectroscopy (FTIR) spectrometer (Agilent Technologies, CA, USA) using potassium bromide pellet methodology in a wavelength range of 400–4000 cm^−1^.

### 2.3 Immobilization of inulinase on mesoporous silica microspheres

For the cross‐linking of inulinase on silica microspheres, 100 µl of inulinase enzyme solution was taken and mixed with 5 mg of the mesoporous silica microspheres. Furthermore, for cross‐linking, 7 mM of glutaraldehyde was added to the reaction solution and then incubated for 12.25 h. After incubation, the mixture was washed three times with phosphate buffer (pH 6.5) by centrifuging at 10,000 rpm for 10 min to obtain the desired cross‐linked inulinase. Subsequently, the samples were assayed for enzyme activity and protein concentration to examine the immobilization efficiency. The activities of the free and immobilized inulinase were assayed according to Kumar et al. [19], by measuring the concentration of reducing sugars released from inulin. The amount of protein in the samples was quantified by Lowry's method [20].

To study the effect of various parameters on the immobilization, the ratio of silica microspheres and inulinase was varied as 0.5:1.0–2.0:1.0 in buffer systems of pH 4.0–9.0. The effect of the concentration of the cross‐linker on immobilization was studied by varying the concentration of glutaraldehyde as 1–10 mM and the reaction mixture was incubated for 0.5–24 h. After immobilization, the immobilized inulinase was characterized by EDS and FTIR analysis with respect to the mesoporous silica support.

To determine the kinetics of the free and immobilized enzyme activities, the hydrolysis of varying concentrations of substrate was studied at the optimum pH and temperature. The kinetic parameters were estimated using Michaelis–Menten (MM) equation.

### 2.4 Hydrolysis of inulin and quantification of fructose

Hydrolysis of inulin was performed using 5% of inulin suspended in phosphate buffer (pH 6.0). The reaction was initiated by the addition of 100 U/mg of immobilized inulinase under the shaking regime of 120 rpm at 50°C [21]. After 3 h of incubation, the amount of fructose released from the reaction was quantified using D‐fructose kit with standard fructose as reference. Similarly, a parallel experiment was performed under the same reaction conditions with 100 U/mg of partially purified inulinase to understand the hydrolysis efficiency of the immobilized enzyme. Furthermore, to comprehend the effect of various process parameters, the key operation parameters in inulin hydrolysis using immobilized inulinase, such as time (0.5–4 h), inulin concentration (1–10%), pH (4.0–9.0) and temperature (40–80°C), were optimized.

### 2.5 Reusability of immobilized inulinase during fructose production

The operational stability of the immobilized inulinase during inulin hydrolysis was studied in a batch system by hydrolyzing 5% inulin with the cross‐linked inulinase (100 U/g) in air‐tight glass vials. The hydrolysis was performed in a buffer system of pH 6.0 for 3 h at 60°C. After every cycle, the mixture was centrifuged and the retrieved immobilized inulinase was washed three times before resuspending it in a fresh substrate solution of 5% inulin.

## 3 RESULTS AND DISCUSSION

### 3.1 Immobilization of inulinase on mesoporous silica microspheres

**FIGURE 1:**
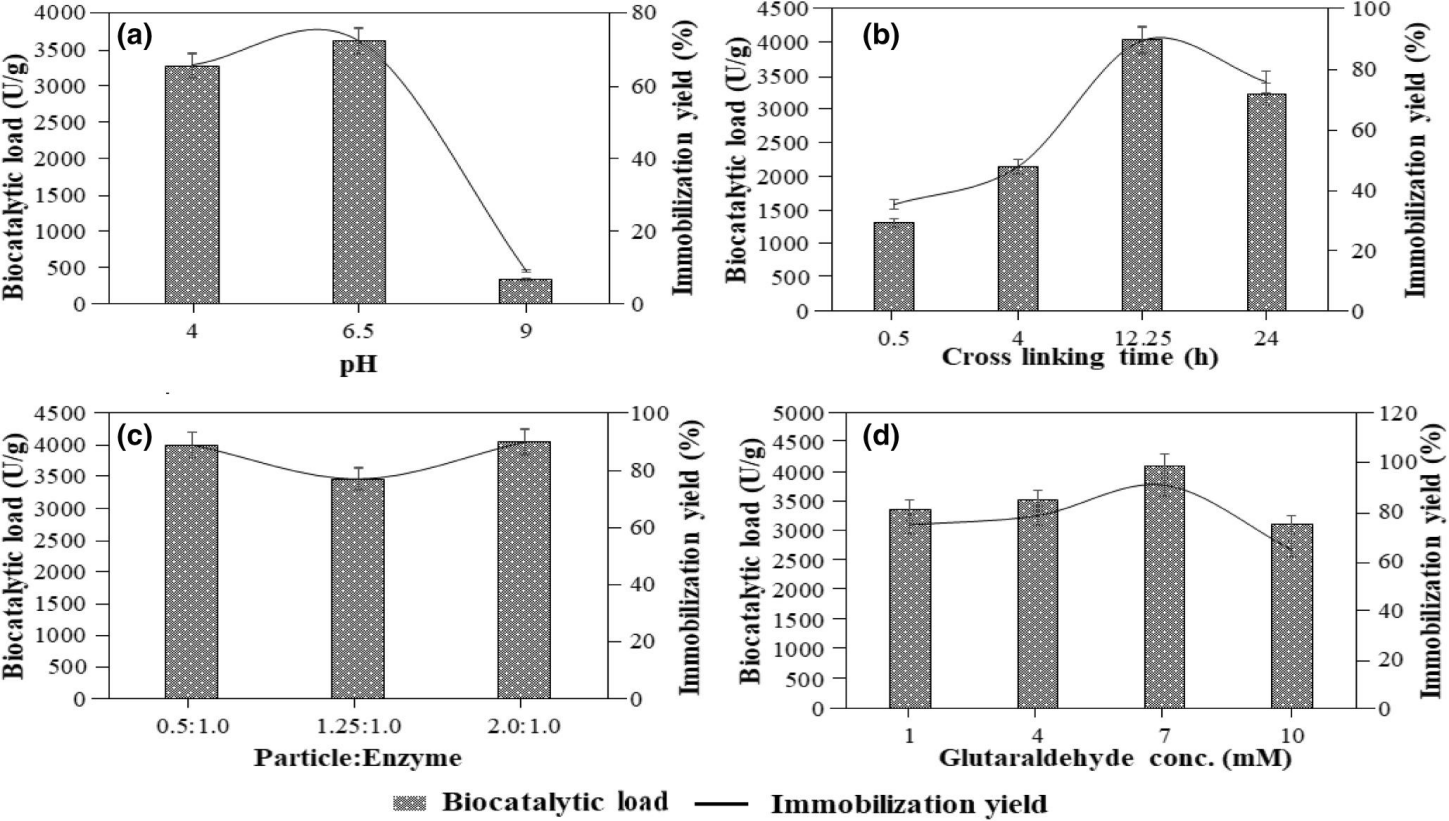
Optimization of (a) pH, (b) cross‐linking time (h), (c) particle:enzyme and (d) glutaraldehyde conc. (mM) during the immobilization of inulinase. The experiments were performed in triplicates and the results are presented as mean average with standard deviation

The partially purified inulinase from *A. brasiliensis* was immobilized on silica microspheres and the effect of various process parameters on immobilization is shown in Figure 1. Since the pH of the system distinctively affects the catalytic activity and structural stability of an immobilized biocatalyst [14], the study was performed in a pH range of 4.0–9.0. It was observed that at pH 4.0, the immobilization yield was 65%, which increased to a maximum of 72.1% at a pH of 6.5. However, increasing the pH to 9.0 was detrimental, which drastically reduced the immobilization yield to 9.1% at the basic pH. Under neutral and acidic conditions, the aldehyde group of glutaraldehyde reacts with the lysine group of the proteins to form Schiff's base, but these bases are highly unstable at acidic pH and hence break down, making near‐neutral pH more favourable, which explained the higher activity of the biocatalyst at near‐neutral pH [15, 22, 23]. In terms of cross‐linking time, after 0.5 h of cross linking, only 35% immobilization yield was achieved which increased to 47.8% after 4 h. This low yield in immobilization could be due to the inefficient binding of the enzyme to the support which hampers the minimum flexibility required for the enzyme activity. On further increasing, the cross‐linking time led to the maximum immobilization yield of 89.4% after 12.25 h. However, a prolonged incubation period beyond 12.25 h was detrimental to the immobilization yield with a high washing loss, possibly due to the lowered flexibility and stability of inulinase. Furthermore, the particle/enzyme ratio determines the extend of immobilization of the enzyme on the support, which contributes to the stability of the immobilized biocatalyst. Here, at a particle/enzyme ratio of 2.0:1.0, the maximum immobilization yield of 90% was obtained. It was observed that a particle/enzyme ratio lower than 2.0:1.0 exhibited higher washing loss which led to lowered immobilization yield [23].

Consecutively, the optimum concentration of the cross‐linker, glutaraldehyde, was studied. At a lower glutaraldehyde concentration, the immobilization yield was low and 74% yield was achieved at a glutaraldehyde concentration of 1 mM. This lowered yield can be explained by the high washing loss due to insufficient binding at a lower glutaraldehyde concentration [15, 24]. However, increasing the glutaraldehyde concentration increased the immobilization yield and a maximum yield of 90.7% was achieved at a glutaraldehyde concentration of 7 mM. Further increasing the glutaraldehyde concentration to 10 mM led to a reduced biocatalytic load despite a considerable immobilization yield of 64.4% which could be due to the loss of enzyme flexibility due to increased rigidity of the enzyme with increased glutaraldehyde concentration, which in turn resulted in limited access of the substrate to the active site [23, 24].

The immobilization yield achieved is similar to that of the previous studies, where Singh and Chauhan [25] achieved an immobilization yield of 84.9% by cross‐linking inulinase on amino (+NH_2_) multiwall carbon nanotubes and Basso et al. achieved an yield of 76% by cross‐linking inulinase on amino Sepabeads [26]. The immobilization yield of inulinase from the present study is compared with various published works in Table S1.

### 3.2 Characterization of mesoporous silica microspheres and immobilized inulinase

**FIGURE 2:**
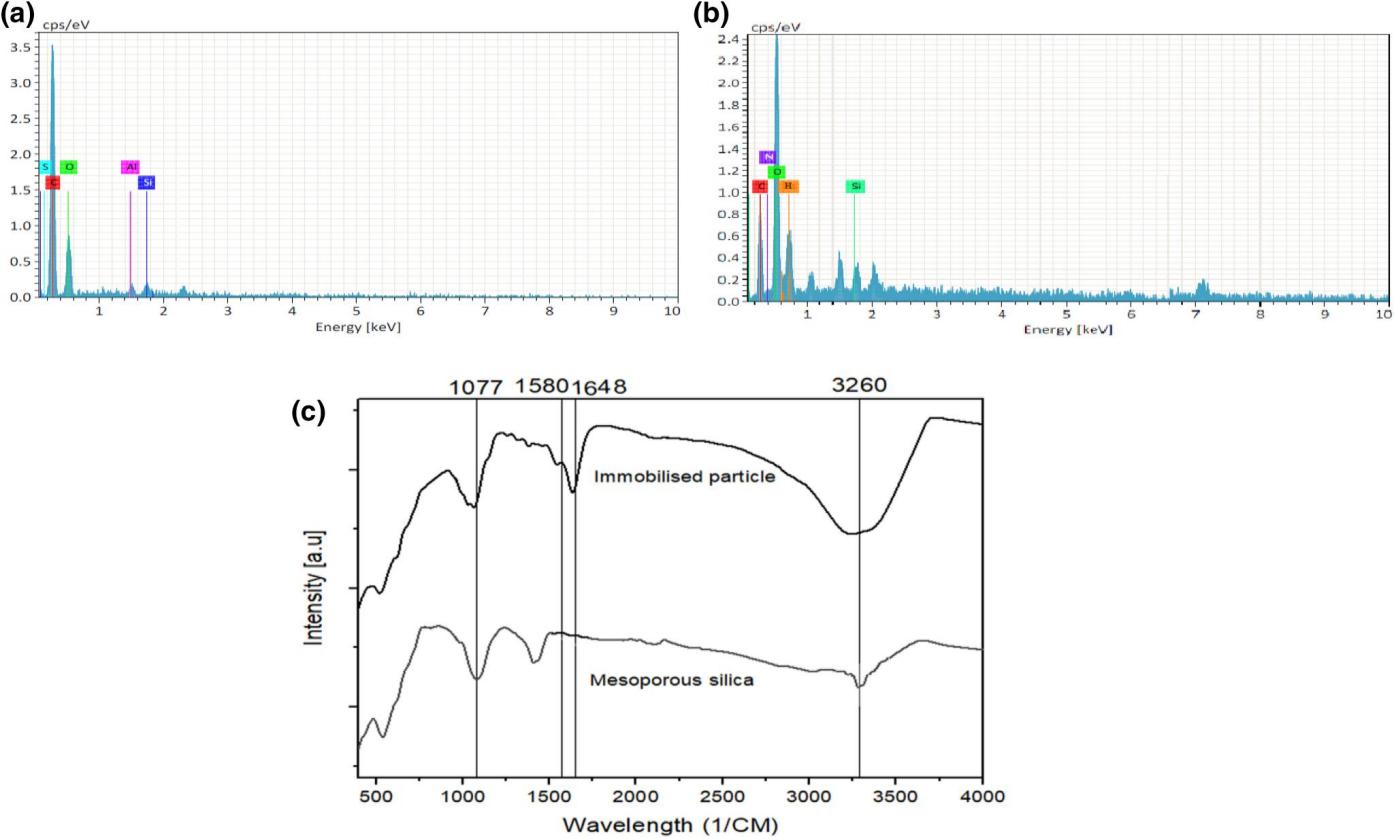
EDS analysis of (a) mesoporous silica microspheres, (b) immobilized inulinase and (c) FTIR of the mesoporous silica microspheres and immobilized inulinase. FTIR, Fourier‐transform infrared spectroscopy.

The shape and surface morphology of the mesoporous silica microspheres was analysed by scanning electron microscopy (SEM; Figure S2). The elemental composition of the mesoporous silica microspheres and immobilized inulinase was confirmed by EDS analysis (Figure 2a,b). The spectrum of mesoporous silica microspheres exhibited a prominent presence of silica at 1.7 keV [27]. Apart from silica, other common elements such as carbon and oxygen were reflected in the mesoporous silica. However, in the inulinase immobilized mesoporous silica particle, a significant amount of nitrogen and hydrogen was noted, which confirms the presence of protein on the surface of the mesoporous silica microspheres. Furthermore, FTIR analysis of the immobilized particles along with the mesoporous silica microspheres were performed to confirm the immobilization of the enzyme (Figure 2c). The presence of silica and oxygen as SiO2 was confirmed by the presence of transmission peak at 3260 cm^−1^. The band at the amide‐I (1648 cm^−1^) and amide‐II (1580 cm^−1^) confirms the presence of the enzyme on the immobilized mesoporous particle. The band at 1077 cm^−1^ is found to be responsible for the Si–O–Si bond stretching [28].

### 3.3 Kinetic parameters

The *K*_m_ value signifies the extent of substrate accessibility to the enzyme which was considered to be increased upon immobilization, due to either structural modification or change in inaccessibility of the active site to the substrate upon immobilization. A similar pattern with a 1.5‐fold increase in the *K*_m_ value for immobilized inulinase when compared to free enzyme was observed, indicating a lower substrate affinity and hence a requirement for a higher substrate concentration for the reactions to progress (Table 1). The apparent *V*_max_ value was significantly reduced by 1.3‐fold for the cross‐linked inulinase when compared to its free counterpart, due to reduction in substrate diffusion to the catalytic site with the increase in *K*_m_ value. The other intrinsic property, *K*_cat_, reflected a 1.0‐fold decrease for immobilized inulinase, which defined the reduction in the number of catalytic cycles each active site underwent per unit time. The reduction in the turnover number can be explained by the cross linking of inulinase on the silica microspheres which led to conformational changes, thus resulting in making the substrate less accessible at the active site of the enzyme [29].

**TABLE 1:** Kinetic parameters of the free and immobilized inulinase

Parameters	Free inulinase	Cross‐linked inulinase
*K*_m_ (mg/ml)	22.4 ± 0.61	34.6 ± 0.42
*V*_max_ (µmol/min)	5.37 ± 0.82	4.19 ± 0.57
*K*_cat_ (s^–1^)	102.5 ± 4.35	97.68 ± 5.19

### 3.4 Hydrolysis of inulin and quantification of fructose

**FIGURE 3:**
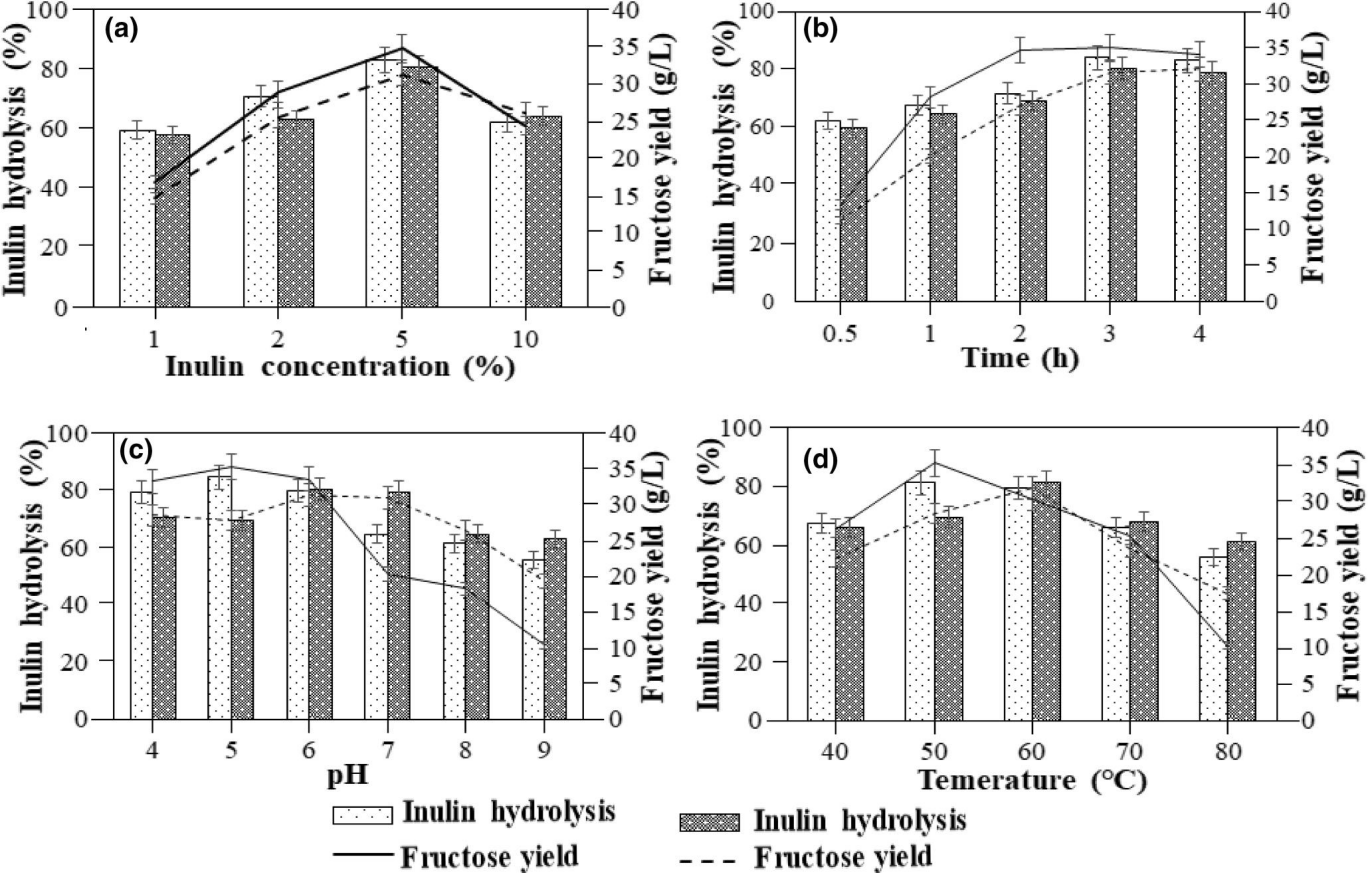
Optimization of (a) inulin concentration (%), (b) time (h), (c) pH and (d) temperature (°C) during inulin hydrolysis. The experiments were performed in triplicates and the result is presented as mean average with standard deviation

The hydrolysis of inulin to HFS was achieved using free and immobilized inulinase under continuous agitation in a batch system, as continuous agitation ensures uniform mixing of the inulin solution [30]. It was observed that after 3 h of hydrolysis of 5% inulin by the immobilized inulinase, a maximum fructose yield of 31.78 g/L was obtained (Figure 3). The concentration of inulin greatly affects the rate of hydrolysis and in the present study, increasing the inulin concentration from 1% to 5% increased the fructose yield and the maximum fructose concentration of 34.8 g/L and 31.3 g/L with the free and immobilized inulinase, respectively, was achieved with 5% inulin. When 5% inulin was used, inulin hydrolysis rate of 83.5% and 80% was achieved using free and immobilized inulinase, respectively. However, increasing the inulin concentration to 10% did not have a beneficial effect on the fructose yield in both the systems as an increased substrate concentration leads to apparent saturation and does not have an opposite effect on the rate of hydrolysis [31]. Furthermore, the hydrolysis time was studied over a time interval of 0.5–4 h for maximum fructose yield, and a maximum fructose yield of 35 g/L and 31.69 g/L with free and immobilized inulinase, respectively, was obtained after a hydrolysis time of 3 h. Subsequently, increasing the hydrolysis time to 4 h increased the fructose yield for both the free and immobilized inulinase system, where the inulin hydrolysis was 85% and 80%, respectively. However, considering the yield of fructose and the economic feasibility of the hydrolysis process, 3 h was considered as the optimized hydrolysis time which was used for further optimization process.

The pH of the system greatly affects the range of inulin hydrolysis [32] and it was observed that at pH 4.0, free inulinase directed a fructose release of 33.25 g/L against 28.47 g/L obtained using immobilized inulinase. Furthermore, increasing the pH gradually enhanced the fructose yield, and a maximum fructose yield of 35.34 g/L was achieved at pH 5.0 for the free inulinase. Whereas, for the immobilized inulinase, the optimum pH was observed to be 6.0 where the fructose release was calculated to be 31.2 g/L. Increasing the pH furthermore towards the basic range led to a decrease in the fructose yield in both the free and immobilized inulinase systems, and a fructose concentration of 10.35 and 19.25 g/L was obtained using free and immobilized inulinase, respectively, at pH 9.0. This decrease in the yield of fructose can be explained by the disorientation of the structure of inulinase at basic pH [10, 22, 33].

Henceforth, during temperature optimization for inulin hydrolysis, it was observed that with the increase in temperature from 40°C, the fructose yield increased using both the free and immobilized inulinase [32].

Considering the hydrolysis of inulin with free inulinase, a maximum fructose yield of 35.14 g/L with 81% inulin hydrolysis was achieved at 50°C. However, increasing the temperature beyond 50°C led to a gradual decrease in the yield of fructose, which reduced to 10.24 g/L at 80°C. Contrarily for the immobilized inulinase system, the fructose concentration gradually increased with temperature and the maximum fructose yield of 31.78 g/L and 70% inulin hydrolysis was obtained at 60°C. This shift to the higher optimal temperature for the immobilized inulinase can be attributed to the increased structural rigidity attained after immobilization [15]. However, increasing the temperature beyond 60°C showed a similar trend like the free inulinase system, where the fructose yield reduced with the increase in temperature and only 17.35 g/L of fructose was obtained at 80°C. At higher temperature, thermal inactivation of the enzyme takes place along with the disintegration of the structural integrity of protein which may lead to the reduced fructose yield at high temperatures [15]. The yield of HFS obtained in this study is similar to the previous works on HFS production by immobilized inulinase and it is tabulated in Table S2.

### 3.5 Reusability of immobilized inulinase during fructose production

**FIGURE 4:**
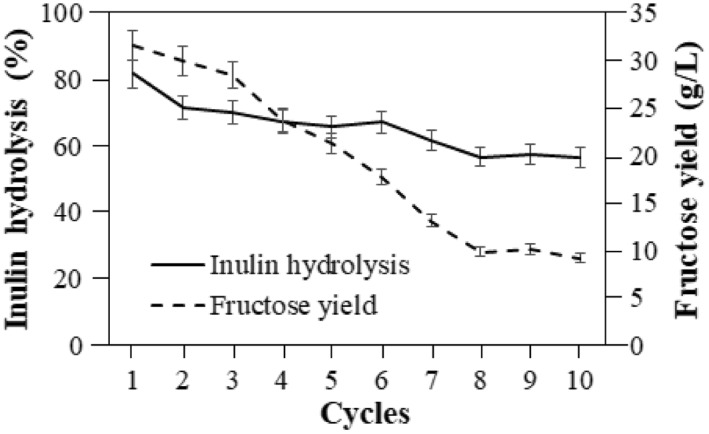
Reusability of immobilized inulinase during inulin hydrolysis. The experiments were performed in triplicates and the result is presented as mean average with standard deviation

The large‐scale application of an immobilized biocatalytic system depends on its operational stability. Thus, the reusability of the immobilized biocatalyst determines the efficiency and feasibility of the process. The reusability of the cross‐linked inulinase was assessed by performing repeated batch cycles of inulin hydrolysis at 60°C. The immobilized inulinase was recycled up to 10 cycles of inulin hydrolysis where 31.5 g/L of fructose was released in the first cycle. With the increase in reusability cycles, the fructose concentration decreased drastically to 17.7 g/L in the 6th cycle, which was possibly due to leaching out of the immobilized enzyme [34]. Prolonged incubation at 60°C adversely affects the thermal stability of the immobilized enzyme [15, 34] and thus, at the 10th cycle, only 9.2 g/L of fructose was released upon inulin hydrolysis (Figure 4). Thus, the reusability of the immobilized inulinase up to 10 cycles of inulin hydrolysis can aid in the possible industrial scale‐up of HFS production for commercialization. Moreover, being environment friendly, this study addresses the industrial need in the design and development of a biocatalytic process by following the main principles of sustainable development—a clean and safe process with maximum efficiency, reuse and recycle along with cost‐efficient as stated by [35].

## 4 CONCLUSION

Inulin represents an inexpensive yet abundantly available raw material for bioprocessing, and the hydrolysis of inulin yields the industrially important HFS. Moreover, the physical and chemical characteristics of mesoporous silica microspheres make them an obvious option as support for enzyme immobilization for enhanced catalyst loading and catalyst retention. Here, inulinase from *A. brasiliensis* was cross‐linked on silica microspheres for the subsequent hydrolysis of inulin, and a maximum fructose concentration of 31.7 g/L was obtained at a pH of 6.5 at 60°C in 3 h. The reusability of the immobilized inulinase up to 10 cycles dictates the possible commercial production of HFS from inulin. Thus, the paradigm shift towards the utilization of low‐cost feedstock for the possible biocatalytic production of high‐valued, industrially important chemicals has presented the greener route for industrial applications.

## CONFLICT OF INTEREST

None.

## Supporting information

Supporting Information S1
