# Supplementary material for: Development of a sustainable route for the production of high‐fructose syrup from the polyfructan inulin
Source: IET Nanobiotechnol. 2021 Mar 22;15(2):149–56. doi: 10.1049/nbt2.12031 (PMC8675771; doi:10.1049/nbt2.12031)
Supplement: Supplementary file 1 — Supporting Information S1 [file NBT2-15-149-s001.docx]

**8. Appendix:**

**Fig. S1: Flow chart describing the immobilization of inulinase on mesoporous silica microspheres.**


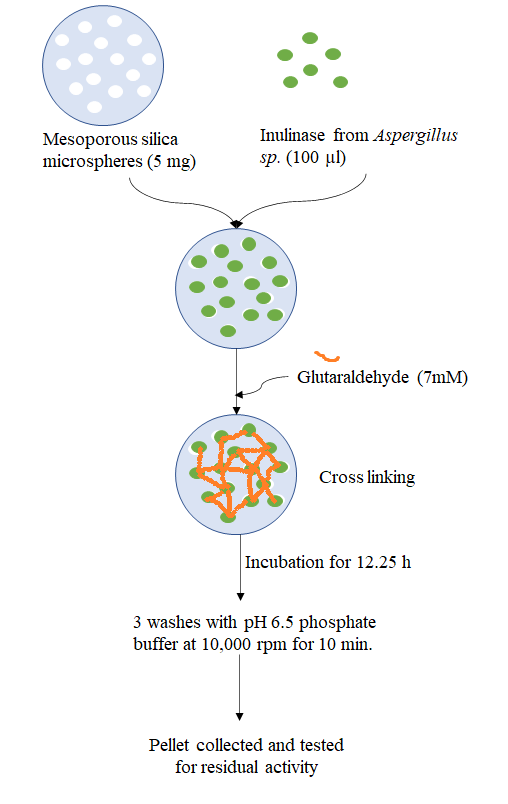


**Fig. S2: SEM image of the mesoporous silica microspheres.**


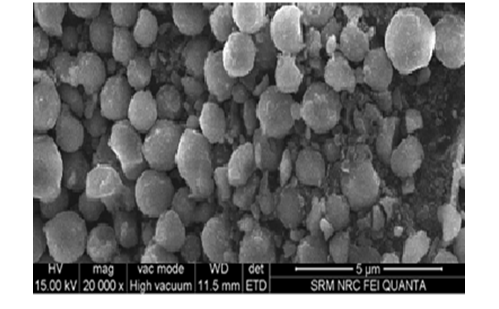


**Table S1: Immobilization of inulinase on various supports**

| **Support** | **Method of preparation** | **Immobilization yield (%)** | **References** |
| --- | --- | --- | --- |
| 3-aminopropyl-triethoxysilane functionalized multiwall carbon nanotubes | Covalent bond | 74.4 | [1] |
| Polyethylene glycol/polypyrrole  multiwall carbon nanotubes | Covalent bond | 85 | [2] |
| Amino Sepabeads | Cross linking | 76 | [3] |
| Amino (+NH_2_) multiwall carbon nanotubes | Cross linking | [4] | [4] |
| Gold magnetic nanoparticles | Covalent bond | 93 | [5] |
| KU-2 ion-exchange resin matrix | Adsorption | 65 | [6] |
| Chitosan-coated magnetite | Cross linking | 81.4 | [7] |
| Glass beads | Covalent bond | 77.2 | [8] |
| Polyurethane foam | - | 49.4 | [9] |
| Chitosan | Cross linking | 66.4 | [10] |
| Mesoporous silica microspheres | Cross linking | 90.7 | Present Study |

**Table S2: Production of high fructose syrup (HFS) by the hydrolysis of inulin by immobilized inulinase**

| **Source of inulin** | **Source of inulinase** | **HFS yield (g/L)** | **References** |
| --- | --- | --- | --- |
| Commercial inulin | Commercial inulinase | 39 | [11] |
| Commercial inulin | Commercial inulinase | 35.8 | [12] |
| Commercial inulin | Inulinase from *Aspergillus sp.* | 6.3 |  |
| Inulin from *Asparagus racemosus* | Inulinase from *Kluyveromyces marxianus* YS-1 | 34 | [13] |
| Inulin from Jerusalem artichoke | Inulinase from *Kluyveromyces*  *Fragilis* | 34 | [14] |
| Commercial inulin | Commercial inulinase | 28.84 | [15] |
| Commercial inulin | Inulinase from *Penicillium oxalicum* BGPUP-4 | 39.6 | [16] |
| Inulin from chicory roots | Commercial inulinase | 36 | [5] |
| Commercial inulin | Inulinase from *Aspergillus niger*  *NCIM 945* | 40.2 | [17] |
| Commercial inulin | Inulin from *Aspergillus brasiliensis* MTCC 1344 | 31.8 | Present study |
